# Test of a Novel *Streptococcus pneumoniae *Serotype 6C Type Specific Polyclonal Antiserum (Factor Antiserum 6d) and Characterisation of Serotype 6C Isolates in Denmark

**DOI:** 10.1186/1471-2334-10-282

**Published:** 2010-09-24

**Authors:** Lotte Lambertsen, Mette B Kerrn

**Affiliations:** 1 Neisseria and Streptococcus Reference Laboratory, Department for Microbiological Surveillance and Research, Statens Serum Institut, Artillerivej, Copenhagen, Denmark; 2 SSI Diagnostica, Statens Serum Institut, Hilleroed, Denmark

## Abstract

**Background:**

In 2007, Park *et al. *identified a novel serotype among *Streptococcus pneumoniae *serogroup 6 which they named serotype 6C. The aim of this study was to evaluate with the Neufeld test a novel *S. pneumoniae *serotype 6C type specific polyclonal antiserum. In addition, serotype 6C isolates found in Denmark in 2007 and 2008 as well as eight old original serotype 6A isolates were characterised.

**Methods:**

In this study, 181 clinical *Streptococcus pneumoniae *isolates from Denmark 2007 and 2008 were examined; 96 isolates had previously been typed as serotype 6A and 85 as serotype 6B. In addition, eight older isolates from 1952 to 1987, earlier serotyped as 6A, were examined. Serotype 6C isolates were identified by PCR and serotyping with the Neufeld test using the novel type specific polyclonal antiserum, factor antiserum 6 d, in addition to factor antisera 6b, 6b* (absorbed free for cross-reactions to serotype 6C) and 6c. All antisera are commercially available and antiserum 6b obtained from the supplier after 1 January 2009 is antiserum 6b*. All serotype 6C isolates were further characterised using multi-locus sequence typing.

**Results:**

When retesting all 96 original serotype 6A isolates by PCR and the Neufeld test, 29.6% (24 of 81) of the invasive isolates in Denmark from 2007 and 2008 were recognised as serotype 6C. In addition, three of eight old isolates originally serotyped as 6A were identified to be serotype 6C. The oldest serotype 6C isolate was from 1962. The serotype 6C isolates belonged to eleven different sequence types (ST) and nine clonal complexes (CC), ST1692 (CC395), ST386 (CC386) and ST481 (CC460) were the predominant types.

**Conclusions:**

We tested a novel polyclonal antiserum 6 d, as well as modified antiserum 6b*, provided a scheme for the serotyping of *S. pneumoniae *serogroup 6 using the Neufeld test and compared the serotyping method with PCR based methods. The two types of methods provided the same results. In future, it will, therefore, be possible to test also serotype 6C in accordance to the standard method for serotyping of *S. pneumoniae *recommended by WHO.

Among all invasive isolates from Denmark 2007 and 2008, serotype 6C constituted 29.6% of the original serotype 6A isolates. The serotype 6C isolates were found to be diverse belonging to a number of different STs and CCs of which most have been observed in other countries previously. Serotype 6C is regarded as an "old" serotype being present among *S. pneumoniae *isolates in Denmark for at least 48 years. The genetic diversity of serotype 6C isolates and their genetic relationship to other serotypes suggested that serotype 6C strains may have arisen from several different independent recombination events involving different parental strains such as serotypes 6A, 6B, 23F and 4.

## Background

In 2007, Park et al. [1] identified a novel serotype among *Streptococcus pneumoniae *serogroup 6 and named it serotype 6C. This novel serotype was identified by the use of monoclonal antibodies and verified by sequencing of the capsule gene locus [1,2]. Interestingly, serotype 6C was found among isolates that were originally identified as serotype 6A using the WHO recommended standard serotyping method of *S. pneumoniae*, the Neufeld test [3]. To our knowledge, it was the first time a novel serotype was found among isolates that were easily serotypeable. In the past, novel serotypes were found among isolates that were non-serotypeable with the available antisera or isolates that showed odd combinations of specific reactions with the antisera.

The genetic difference between serotypes 6A and 6C is the *wci*N gene [2] encoding a transferase resulting in an altered composition of the polysaccharide capsule. This gene may be used to distinguish serotype 6A from 6C and as soon as the news concerning this novel serotype was out, many laboratories started testing for serotype 6C using PCR [4-7]. Subsequently, serotype 6C has been detected among invasive isolates [8] as well as non-invasive [5] and carriage isolates [9] and to date serotype 6C has been reported in the following countries: Canada, the U.S., Mexico, Brazil, the Netherlands, Portugal, South Africa, Korea, China and Australia [10,6,8,9,11]. These countries represent most continents. Serotype 6C is, therefore, not considered a rare serotype and it is expected that it will be found in all parts of the world. For that reason, it is of interest to be able to identify serotype 6C using the Neufeld test and typespecific polyclonal antisera as is the case for the 90 other serotypes.

The aim of this study was to evaluate with the Neufeld test a novel *S. pneumoniae *serotype 6C type specific polyclonal antiserum raised in rabbits. This novel antiserum has been commercially available since 1 January 2009. In addition, serotype 6C isolates found in Denmark in 2007 and 2008 as well as eight older serotype 6A isolates were characterised.

## Methods

### Bacterial isolates

In this study, 181 clinical *Streptococcus pneumoniae *isolates (one isolate per case) from 2007 and 2008 were examined. Of these isolates, 96 had previously been typed as serotype 6A and 85 as serotype 6B. The isolates were received in the reference laboratory from Danish clinical microbiological laboratories. Ethical approval was not needed for this study. The sample origin of the isolates is shown in Table 1. In addition, eight older isolates from the period 1952 to 1987, which had earlier been serotyped as 6A were examined. As positive controls, we used serotype 6C isolates identified in two other countries, one isolate from the Netherlands (Peter Hermans, Nijmegen, The Netherlands) and three isolates from the U.S. (Michael Jacobs, Cleveland, Ohio).

**Table 1:** Overview of analysed *S. pneumoniae *serotype 6C

Serotype	6A% (n)	6B % (n)	6C % (n)
**Invasive isolates**			

blood	84.2 (48)	90.6 (58)	79.2 (19)
cerebrospinal fluid	14.0 (8)	6.3 (4)	16.7 (4)
other sterile site	1.8 (1)	3.1 (2)	4.2 (1)
Proportion of serogroup 6	39.3 (57)	44.1 (64)	16.6 (24)
6C of original 6A			29.6 (24 of 81)

**Non-invasive isolates**			

eye	25.0 (3)	9.5 (2)	
ear	33.3 (4)	38.1 (8)	100 (3)
other non-sterile site	41.7 (5)	52.4 (11)	
Proportion of serogroup 6	33.3 (12)	58.3 (21)	8.3 (3)
6C of original 6A			20.0 (3 of 15)

**All isolates**			

Proportion of serogroup 6	38.1 (69)	47.0 (85)	14.9 (27)
6C of original 6A			28.1 (27 of 96)

Analysed *S. pneumoniae *serotype 6C isolates compared to other serogroup 6 isolates from Denmark 2007 and 2008.

Isolates were grown on 10% blood agar plates (SSI Diagnostica, Hilleroed, Denmark) at 36°C in air with 5% CO_2 _and stocks of isolates were stored at -80°C in beef broth with 10% glycerol (SSI Diagnostica). Bacterial chelex-solution lysates were used as DNA templates for PCR.

All isolates were tested for susceptibility to antimicrobial agents as described in the DANMAP 2008 report http://www.danmap.org.

### PCR for identification of serotype 6C

For PCR1, primers 5106 (5-TACCATGCAGGGTGGAATGT) [2] and 3101 (5-CCATCCTTCGAGTATTGC) [12] were used. For PCR2, primers 6A-N-6195 (5-TTGGACAATCTGGAAAGATATTG) and 6A-N-7642 (5-GCTTTTTTAGCAGGCGACATAG) were used (numbers in the PCR2 primer names refers to the 5'annealing site of the primer to sequence [GenBank: EF538714]). PCR was performed using a 25 μl PCR mix with 12.5 μl HotstarTaq Mastermix (Qiagen, Hamburg, Germany), 0.5 mM additional MgCl_2_, 0.2 μM of the relevant primers and 2 μl DNA-template. The PCR programme used was; 15 min at 95°C, 35 cycles of 30 sec at 95°C, 30 sec at 50°C (52°C for PCR2) and 90 sec at 72°C. The PCR was finalised by 10 min at 72°C. The presence and quality of the PCR fragments was tested by gel-electrophoresis on 2% E-gels (Invitrogen A/S, Taastrup, Denmark).

PCR1 resulted in PCR fragment sizes of 1,45 kb from serotype 6A and 1,25 kb from serotype 6C. PCR2 resulted in an 2,00 kb PCR fragment from serotype 6A and an 1,80 kb fragment from serotype 6C.

### Sequence determination of *wci*N

Excess primers and nucleotides in the solution containing the PCR fragments from PCR1 or PCR2 were inactivated with ExoSap IT^® ^as described by the manufacturer (USB Corporation, Cleveland, USA) before sequencing. Both strands of the PCR fragments were sequenced using 0.2 μM of the relevant primers, BigDye Terminator 3.1 Cycle Sequencing Kit (Applied Biosystems, Foster City, USA) and an ABI-prisme 3100 Genetic Analyzer (Applied Biosystems) as recommended by the manufacturer.

### Serotype determination and test of serotype 6C type specific antisera

Serogroup determination was performed by agglutination test using Pneumotest Latex (SSI Diagnostica). Briefly, a culture of each isolate was grown in serum broth (SSI Diagnostica) overnight at 36°C in air with 5% CO_2_. An aliquot of 10 μl culture and 10 μl of latex suspension were placed next to each other on a cardboard reaction card, mixed and spread with a wooden tooth-stick followed by rocking the card from side to side. Each isolate was tested for reactions in all latex pools at the same time. Positive reactions showed as clear agglutination reactions within 10-20 sec. In the case of serogroup 6, these were positive reactions in latex pool B and Q. Serotype determination was performed with the Neufeld test using *S. pneumoniae *type specific rabbit antisera as recommended by the manufacturer (SSI Diagnostica). In this case, the factor antisera used were 6b, 6b* (absorbed free for cross reactions to serotype 6C), 6c and the novel antiserum 6 d. Briefly, isolates were grown on 10% blood agar plates overnight at 36°C in air with 5% CO_2_. A small amount of bacteria from the plate was suspended in an aliquot of 1-2 μl saline placed on a microscope slide. Finally, 1-2 μl factor antiserum was added to the bacterium-saline suspension with an 1 μl plastic inoculation needle. A glass cover slip was placed on the mixture, a drop of immersion oil added and the bacteria were inspected in a light microscope with 100× magnification, phase-contrast and a green filter. Each isolate was tested for reaction with all factor antisera within the group at the same time. A positive reaction was observed as a swelling of the bacterium and capsule, often agglutination also occurred.

### Multi locus sequence typing (MLST)

Sequence types were determined as described at http://spneumoniae.mlst.net/.

### Data analysis

All sequences were analysed using the software BioNumerics (Applied Math, Sint-Martens-Latem, Belgium) and sequence types identified with the information provided at http://spneumoniae.mlst.net/. Clonal relationships of sequence types and predictions of founders were analysed using the programme eBURST and the complete MLST *S. pneumoniae *database at http://spneumoniae.mlst.net/. Sequences of *wci*N were compared to the sequence of *S. pneumoniae *CHPA388 [GenBank: EF538714], sequence and amino acid numbers refer to that sequence.

Predictions of the protein secondary structure and the amino acid accessibility was analysed using the programme NetSurfP at http://www.cbs.dtu.dk/services/NetSurfP/[13].

## Results

### Genetic identification of *S. pneumoniae *serotype 6C isolates by PCR and sequencing of the *wci*N gene

Of all 96 original serotype 6A isolates from 2007 and 2008, 28.1% (n = 27) were tested to be serotype 6C by both PCR1 and PCR2 described in Methods. The PCR fragments from all isolates had the expected sizes and were similar to those from the positive controls of serotype 6C isolates identified in two different countries.

The coding region of the *wci*N genes of all 27 PCR detected 6C isolates were 100% identical among the isolates. The genes showed 99.7% similarity to the *wci*N gene of 6C [GenBank: EF538714] published by Park et al 2007 and 99.9% similarity to the four other published sequences [GenBank: EF538716, EF538717, EF538715 and EF538718]. Compared to [GenBank: EF538714] sequence, the nucleotide changes were T65A, C541A and G677T. These changes did result in amino acid changes of the WciN protein of F22Y, H181N and G226V, however, these changes did not alter the predictions of the secondary structure and the amino acid accessibility analysed using the programme NetSurfP at http://www.cbs.dtu.dk/services/NetSurfP/[13]. Using this programme, amino acids 22 and 181 were most likely located in alpha-helixes and amino acid 226 was most probably part of a beta-strand; all three amino acids were predicted to have low relative and absolute surface accessibility.

### Test of *S. pneumoniae *serotype 6C type specific polyclonal factor antiserum 6d

A novel type specific rabbit antiserum, named factor antiserum 6 d, was tested in addition to the existing factor antisera 6b and 6c (Table 2) on all original serotypes 6A (n = 96) and 6B (85) isolates. Using the Neufeld test, all serotype 6A isolates detected by PCR (n = 69) showed positive and specific reactions with factor antiserum 6b. All serotype 6B isolates showed positive and specific reactions with factor antiserum 6c. All serotype 6C isolates detected by PCR (n = 27) showed positive and specific reaction with factor antiserum 6d; however, in addition the serotype 6C isolates showed positive and specific reactions with factor antiserum 6b. Therefore, a modified factor antiserum 6b* absorbed free for cross reactions to serotype 6C was produced and tested. This antiserum should react specifically with serotype 6A but not 6C. All serotypes 6A and 6C isolates were tested and only serotype 6A isolates showed positive and specific reactions with factor antiserum 6b*. The resulting serotyping scheme for serogroup 6, including the novel factor antiserum 6 d and the modified antiserum 6b*, is shown in Table 2.

**Table 2:** Serotyping scheme for *S. pneumoniae *serogroup 6

Serotype	Factor serum				
	**6a**	**6b**	6b*	**6c**	**6d**

**6A**	x	x	x		
**6B**	x			x	
**6C**	x	x			x

* absorbed with 6C

Serotyping scheme for *S. pneumoniae *serogroup 6 using the Neufeld test and polyclonal factor antisera. Scheme for determining the serotype from the pattern of positive reactions (x) between *S. pneumoniae *and type specific polyclonal factor antisera.

### Origin of serotype 6C isolates from 2007 and 2008 compared to other serogroup 6 isolates from Denmark

Upon retesting original serotype 6A isolates by PCR and the Neufeld test, a proportion of 29.6% (24 of 81) of the invasive isolates were serotype 6C (Table 1). These isolates were from 23 patients with a mean age of 53.5 years (range 0 to 92 years). In the same period of time from the same geographic area, there were 56 serotype 6A and 62 serotype 6B isolates (Table 1). The serotype 6A isolates were from patients with a mean age of 63.0 years (range 0-91 years) and the serotype 6B isolates were from patients with a mean age of 58.9 (range 0-99). Of the 6C isolates, 48% were from females compared to 54% and 56% of serotype 6A and 6B isolates, respectively.

All invasive isolates were tested for susceptibility to the two antimicrobial agents penicillin and erythromycin, 3.7% (n = 2) of serotype 6A, 6.5% (4) of serotype 6B and 4.3 (1) of serotype 6C were penicillin non-susceptible and 3.7% (n = 2) of serotype 6A, 9.7% (6) of serotype 6B and 13% (3) of serotype 6C were erythromycin resistant.

### Clonal relation of serotype 6C isolates from Denmark using multi locus sequence typing and antimicrobial resistance pattern

Among all serotype 6C isolates, eight different sequence types (STs) were found. The most prevalent STs were ST1692 (n = 10), ST386 (6) and ST481 (5) (Table 3). Most serotype 6C isolates were susceptible to the antimicrobial agents tested and the non-susceptible isolates were found to belong to two STs, ST386 and ST1379 (Table 3).

**Table 3:** Sequence types (STs) and clonal complexes (CCs) of serotype 6C

2007-2008 is olates					
**Number of isolates, n**	**ST**	CC (founder)*	**ST Antimicrobial resistance profile (n)**	**ST has been found in serotypes (countries)^#^**	**CC has been found in serotypes (countries)^#^**

10	1692	395		6A (UK, Switzerland) 6C (Portugal)	6A (UK, U.S.) 6C (Portugal, Italy)
6	386	386	PEN, ERY, CLI, TET (3) ERY, CLI, TET (3)	6B (Portugal, Italy, Lebanon, Germany)	6B (Portugal, Italy, Lebanon, Germany)
5	481	460		6A (Finland)	6A (UK, U.S., France)
3	1379	1600	ERY (1)	6A (Sweden, U.S.) 6C (U.S., Australia)	6A (UK, U.S.)
1	398	473		6A (UK)	6A (UK, U.S., France, Greece, Australia, Germany) 6B (Australia, Hungary) 6C (U.S.)
1	600	600		6A (Polan) 6C (Australia)	6A (Polan) 6C (Australia)
1	1714	395		6A (UK) 6C (Portugal) 23F (Norway)	6A (UK, U.S.) 6C (Portugal, Italy)

**Old isolates**					

1	2185	2185		6A (Polan) 6C (Portugal)	6A (Polan) 6C (Portugal)
1	3029	3304		6A (U.S.)	4 (The Gambia)
1	new1				

* in accordance to eBURST using the complete MLST database and a minimum similarity of six identical loci

^#^information in accordance to the http://www.mlst.net accessed 26. June 2010

Sequence types (STs) and clonal complexes (CCs) of serotype 6C isolates from Denmark in relation to those found previously in other countries.

To evaluate the clonality of the serotype 6C isolates, the founder of the clonal complex (CC) for each ST was predicted using eBURST. The eight STs were found to belong to seven different CCs (Table 3). By this method, the serotype 6C isolates were found to be diverse.

### Test of old serotype 6A isolates

Eight old isolates originally serotyped as serotype 6A isolates were also tested. Three of them were typed to be serotype 6C by the PCR methods and the Neufeld test using the novel antisera. These three isolates were from the years 1962, 1968, and 1987. The *wci*N gene sequences were identical to the 6C isolates analysed from 2007 to 2008, except for one of the isolates that had a silent A7298G sequence change. The three isolates all had different STs, ST3029, ST2185 and STnew1 belonging to three different CCs.

## Discussion

This study compared two different methods to identify serotype 6C *S. pneumoniae; *a phenotypic method, the Neufeld test, based on the use of a novel polyclonal factor antiserum 6 d specific for serotype 6C and a genotypic method based on PCR identification of the *wci*N gene size difference between serotypes 6C and 6A. The two methods provided the same results when testing 96 original serotype 6A isolates.

In addition to the novel antiserum 6 d, antiserum 6b that showed specificity to both serotypes 6B and 6C was altered to antiserum 6b* specific only to serotype 6B and not to serotype 6C. These novel antisera together with the known sera, should provide a sufficient panel of antisera to be able to distinguish the currently known serotypes of serogroup 6. All antisera are commercially available and antiserum 6b obtained from the supplier after 1 January 2009 is antiserum 6b*.

With this study we have shown that it was possible to produce useful polyclonal antisera to be able to distinguish serotype 6C from the other serotypes within serogroup 6. The specificity of the factor serum 6 d was in agreement with a recent validation study of this factor serum performed by a different research group [14]. However, in that study, factor serum 6b and not 6b* was used, which is why they observed serotype 6C to be positive in both factor sera 6 d and 6b.

Very recently, a research group at CDC also showed that it was possible to produce a specific polyclonal antiserum to distinguish serotype 6C from other serogroup 6 isolates following the same strategy as described here; their antisera are, however, currently not commercially available [15].

One different phenotypic method based on monoclonal antiserum identifying one specific epitope for the identification of serotype 6C has been reported [1]. In contrast, several genetically based methods based on the identification of the *wci*N gene or sizes of specific PCR fragments have been reported before [2,7,10,6].

With the discovery of serotype 6C, it became clear that new technology, especially genetically based methods, will change the number of known serotypes and the division of groups into serotypes. It also became clear that it is possible to use different methods for determining a serotype. More methods provide possibilities for more laboratories to perform serotype determination of isolates, which is of high value when estimating and evaluating vaccine efficacies and vaccine failures. Different methods for serotype determination will complement each other, but all methods should be compared to one international standardised reference method.

The international reference method and the WHO recommended method for the serotyping of *S. pneumoniae *is the Neufeld test and the use of type specific polyclonal antisera. With the production of the novel antisera reported here, the Neufeld test can continue to be the international reference method.

In 2007 and 2008, serotype 6C constituted 28.1% (27 of 96) of all original serotype 6A isolates received in the Danish national reference laboratory. Among all invasive isolates from the same time period, serotype 6C constituted 29.6% (24 of 81) of the original serotype 6A isolates. This corresponds to 1.2% of all invasive *S. pneumoniae *isolates and an incidence of 0.22 per 100,000 persons. This proportion is at the same level as observed in other studies. Among isolates in Cleveland, the U.S., from 1999 to 2007, 6C was observed to constitute 26% of the previous serotype 6A isolates [5], among invasive isolates from Brazil 1996 to 2007, 34% of previous serotype 6A isolates were found to be serotype 6C [10], and among Australian invasive isolates previously defined as serotype 6A, serotype 6C constituted 31% [7]. In contrast, serotype 6C was found to constitute only 5% of invasive isolates from children in South Africa in 2005-2006 [11] and only 0.3% among previously defined 6A carriage isolates from Dutch children in 2002 [4]. These studies showed that serotype 6C is widely spread in the world and that it may constitute a higher or lower proportion of the previously defined serotype 6A isolates. The proportion of serotype 6C compared to the other serotypes of serogroup 6 as well as the proportion of serotype 6C compared to all serotypes is expected to change with time and may change differently in different areas with use of vaccines and antimicrobial agents as factors that can provoke serotype distribution changes. Such changes within serogroup 6 have already been observed by the Active Bacterial Core Surveillance Team in the U.S. They tested 16% invasive isolates of original serotype 6A in 1999 to be serotype 6C; however, this changed to 65% and 69% in 2006 and 2007, respectively. The change corresponded to a change in the incidence of 6C invasive pneumococcal disease from 0.22 in 1999 to 0.58 in 2007 [6]. The dramatic change was related to the introduction of the 7-valent pneumococcal vaccine in 2000 causing a decline in the incidence of both serotype 6B and 6A cases, but an increase in the proportion of serotype 6C cases [ref [6]]. This was probably observed, as the vaccine protects against invasive infections with serotype 6B, but in addition the vaccine has been shown to have a high level of cross-protection against serotype 6A but not 6C [16]. Similar changes in the distribution of serotypes within serogroup 6 can be expected in Denmark in the future, as the vaccine was introduced in 2007.

The sample origin (sample material, age and sex of person) and antibiotic resistance level of serotype 6C were quite similar to that found for the other serogroup 6 isolates. The proportion of antibiotic non-susceptible 6C was low compared to observations done in the U.S. [5,6,17], but that may also change as observed by the Active Bacterial Core Surveillance Team in the U.S. after introduction of the vaccine [ref [6]].

Among all Danish serotype 6C isolates from 2007 and 2008, ten different STs belonging to nine CCs were found and among the three old 6C isolates three different STs and CCs were found. The serotype 6C isolates in Denmark were, therefore, regarded as diverse. Except for ST386 and STnew1, all of the serotype 6C STs had been identified before in relation to serotype 6A from a number of different countries (Table 3). It would be interesting to re-analyse those serotype 6A isolates as they may turn out to be serotype 6C with the current knowledge. Thus ST481 and CC460 had only been related to serotype 6A, they may, therefore, be true serotype 6A and not 6C if re-analysed. Four of the STs were previously found in relation to serotype 6C, all from geographically distant countries like Portugal, Australia and the U.S. (Table 3). One ST, ST386 had previously only been found in relation to serotype 6B in several countries (Table 3). All of the CCs, except for CC386 and CC3304, had also been observed in a number of countries in relation to serotypes 6A or 6C (Table 3). CC386 had only been found in serotype 6B isolates and in addition to 6A and 6C, CC473 had also been observed in serotype 6B. CC395 had in addition to serotypes 6A and 6C also been observed in serotype 23F whereas CC3304 was previously only observed in serotype 4 (Table 3). All of these observations confirm the suggestion by Carvalho et al. 2009 that serotype 6C strains arose from independent recombination events. Thus, in contrast to that study suggesting these involved parental strains of serotypes 6A and 6C, this study suggests that these events involve at least serotypes 6A, 6B and possibly also serotypes 23F and 4 as parental strains. With this study, it was also confirmed that serotype 6C is an "old" serotype being present among *S. pneumoniae *isolates in Denmark for at least 48 years. This time span would allow the possibility that 6C strains may have arisen from several different independent recombination events involving different parental strains.

## Conclusions

In this study we tested a novel polyclonal antiserum 6 d, as well as a modified antiserum 6b*, provided a scheme for serotyping of *S. pneumoniae *serogroup 6 using the Neufeld test and compared the serotyping method with PCR based methods. The two types of methods provided the same results. In the future, it will therefore be possible to test also serotype 6C in accordance to the WHO recommended standard method for serotyping of *S. pneumoniae*.

Among all invasive isolates from Denmark 2007 and 2008, serotype 6C constituted 29.6% of the original serotype 6A invasive isolates. The serotype 6C isolates were found to be diverse belonging to a number of different STs and CCs of which most had been observed in other countries previously. Serotype 6C is regarded as an "old" serotype with the presence among *S. pneumoniae *isolates in Denmark for at least 48 years. The genetic diversity and relationship of the analysed 6C strains suggest that serotype 6C strains may have arisen from several different independent recombination events involving different parental strains such as serotypes 6A, 6B, 23F and 4.

## Competing interests

The authors declare that they have no competing interests.

## Authors' contributions

LL and MBK both contributed to this study by the conception and design of the study, MBK produced and provided the novel antisera, LL managed all laboratory work, collected data and did data analyses. LL drafted the manuscript and both LL and MBK revised the manuscript critically. Both authors have approved the final manuscript.

## Pre-publication history

The pre-publication history for this paper can be accessed here:

http://www.biomedcentral.com/1471-2334/10/282/prepub
